# Establishment of canine models of lunatomalacia through liquid nitrogen freezing

**DOI:** 10.3892/etm.2013.904

**Published:** 2013-01-17

**Authors:** QISHUN HUANG, QIANG FU, HUAIYUAN ZHENG, MENG GAN, YUXIONG WONG, ZHENBING CHEN

**Affiliations:** Department of Hand Surgery, Union Hospital Affiliated to Tongji Medical College, Huazhong University of Science and Technology, Wuhan, Hubei 430022, P.R. China

**Keywords:** animal models, liquid nitrogen freezing, lunate, lunatomalacia

## Abstract

The aim of this study was to investigate the feasibility of establishing dog models of lunatomalacia through liquid nitrogen freezing. Twelve adult crossbred dogs were divided into three groups. Unilateral lunates were peeled off the parenchyma and frozen to result in avascular necrosis. They were observed dynamically through X-ray, computed tomography (CT) and magnetic resonance imaging (MRI). Furthermore, gross and histomorphological observations of samples were performed. Disseminated punctate hyperintense images and abnormal manifestations were detected, respectively. At 12 weeks after surgery, uneven bone density of the lunate and a flattened lunate of irregular shape were detected. A large area of irregular hypointense foci and hyperintensity was observed. Gross sample observation revealed a large area of dead bone. A decrease in the density of the trabecular bones and several vacant bone lacunas were visible. Liquid nitrogen freezing is a successful and reliable method for preparing animal models of lunatomalacia.

## Introduction

Lunatomalacia, one of the main causes of wrist pain, is a disease characterized by cystic change, fragmentation and progressive collapse of the lunate (1–3). Lunatomalacia was first reported by Peste in 1843. In 1910, Robert Kienböck formulated a classic description of its clinical and X-ray manifestations, for which it was also named Kienböck’s disease (4). More than 100 years have passed since the first report on lunatomalacia; however, the origin of this disease remains unclear and no theory satisfactorily explains its original factors and pathological process. To date, a number of factors have been thought to correlate with the pathogenesis of lunatomalacia. These factors include vascular factors, ulnar minus variation, morphological changes of the lunate and blood hypercoagulable states due to systemic factors, a decrease in arterial perfusion and venous blood stasis, application of corticosteroids, sickle red blood cell disease, cerebral palsy and septicemic embolism (5–8). These factors require different treatment methods. For early-phase treatment of lunatomalacia, non-operative approaches and bed rest are mainly taken. For aggressive-phase treatment, various surgical procedures may be adopted, each having its own advantages and disadvantages (9–11). Although different surgical routes offer multiple choices for lunatomalacia treatment, no criteria currently exist for determination of a preferred method of treatment (2). With the exception of conservative treatments, all the methods to treat the lunatomalacia by surgical procedures are substitution therapies, in which one trauma is adopted for another. They are not specifically based on the origin of lunatomalacia or its pathological changes.

To date, studies on animal models of lunatomalacia remain rare. Accordingly, this study aimed to provide an experimental basis for the clinical treatment of lunatomalacia by creating dog models of lunatomalacia through liquid nitrogen freezing.

## Materials and methods

### Animals

A total of 12 adult healthy crossbred dogs weighing 15–20 kg were selected. The surgical procedures were approved by the Institute of Animal Ethics of Tongji Medical College.

### Sample perfusion

The blood supply sources for the lunate were identified using the emulsion perfusion method. The upper extremities were taken from fresh dog cadavers and 1 liter heparin saline (at a ratio of 12,500 units heparin to 200 ml physiological saline) was injected into the axillary artery until a colorless liquid was observed to flow out from the veins. A red emulsion (red ink and white emulsion, 1:8) was then injected into the axillary artery until a red liquid was observed to flow out from the proximal veins. The samples were placed in a refrigerator for 24 h and then taken out.

### Applied anatomy: constitution of carpal bones

The carpal bones of dogs are composed of two bones in the proximal row and four in the distal row. The radial carpal bone in the proximal row is the lunate (or scapholunatum). Dogs are often used as animal models for studies on lunate or scaphoid diseases due to the similarity of their anatomical position and the biomechanic relationships in the constitution of the carpal bones to those of humans.

### Distribution of blood vessels

The axillary artery was traced until the elbow, where it diverged into the radial and ulnar arteries at the proximal end of the forearm. The radial and ulnar arteries spread off in branches at the wrist to form volar and dorsal nets. Tiny branches from the nets extended into the lunate. No blood vessels were observed on the articular surface of the lunate.

### Model preparation

Skin preparation was performed on a unilateral upper extremity. After administration of an intravenous anesthetic with 3% pentobarbital, an S-shaped incision was made on the dorsum of the radial carpal joint in a sterile condition to expose the wrist joint completely. The joint capsule was cut open and the lunate was isolated. The volar tissues were stored. A two-ply sterilized rubber tissue was used to separate the lunate from its surrounding tissues. The lunate was frozen in liquid nitrogen for 10 min and then warmed to room temperature. After three cycles of freezing and warming, the joint capsule, ligaments and skin were sutured and then fixed with plaster. The dogs were fed on normal food and the raising conditions were kept the same as those before surgery. After 3 weeks, the plaster was dismantled and the stitches were removed. The animals were observed at 3, 6 and 12 weeks after surgery. At each observation time point, four dogs were anesthetized and studied iconographically. After the dogs were sacrificed, their bilateral lunates were removed for gross and histomorphological examinations.

### Observational indices and methods: general conditions

The weight, mental state, coat color, gait and wound of the dogs were observed.

### X-ray detection

Lateral radiographs of the bilateral lunates were obtained at 3, 6 and 12 weeks after surgery. The experimental lunate was compared with the control at each observation time point to determine the changes in the density of the lunate and bone destruction following surgery. The results were also analyzed based on the Lichtman staging criteria (12).

### Computed tomography (CT) scanning

Bilateral lunates were scanned by CT and reconstructed three-dimensionally to observe changes in the morphology, sclerotin and density of the lunate following surgery.

### Magnetic resonance imaging (MRI) detection

MRI detection was performed using an eight-channel wire coil to observe changes in the signals and morphology of the lunate following surgery.

### Visual observation

The shapes, sizes, articular surfaces, color and densities of the bilateral lunates were observed and compared visually.

### Histomorphological observation

The lunate was cut open along the coronal plane, fixed with 10% formaldehyde solution, decalcified with 5% nitric acid, dehydrated gradiently with routine ethanol and then embedded with paraffin. Sections were cut and stained with hematoxylin and eosin.

## Results

### General observation

Following surgery, the dogs presented a negative mental state. The appetite, weight and glossiness of the coat of the dogs decreased. Furthermore, subcutaneous fat was observed. Four weeks after surgery, they began to support their weight using the forelimb on the side utilized for preparing the model.

### X-ray detection

**Figure 1.**
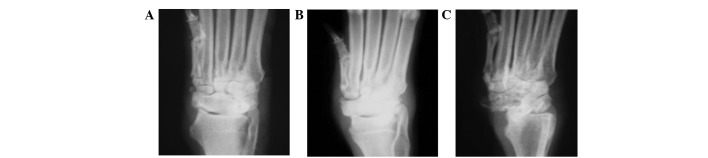
X-ray images. No marked changes in sclerotin were found in weeks (A) 3 or (B) 6 after surgery. (C) In week 12 after surgery, the density of the lunate appeared uneven, the capsular space flattened and the lunate presented an irregular shape with an unsmooth articular surface.

No marked changes in sclerotin were identified at 3 and 6 weeks after surgery. However, abnormalities were observed at 12 weeks after surgery. The density of the lunate became uneven, the capsular space flattened and the lunate presented an irregular shape with an unsmooth articular surface (Fig. 1).

### CT scanning

**Figure 2.**
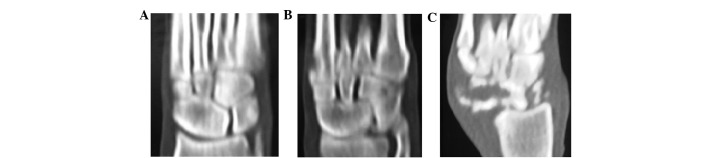
Images by computed tomography (CT) scanning. No marked abnormalities were detected by CT three-dimensional reconstruction in weeks (A) 3 or (B) 6 after surgery. (C) In week 12 after surgery, a damaged articular surface and discontinuous bone cortex were identified. The structure of the trabecular bones disappeared and hypointense foci of irregular morphology appeared in the pulp chamber.

No obvious abnormalities were detected by CT three-dimensional reconstruction at 3 or 6 weeks after surgery. Damaged articular surface and discontinuous bone cortex were apparent at 12 weeks after surgery. The structure of the trabecular bones disappeared and hypointense foci of irregular morphology appeared in the pulp chamber (Fig. 2).

### MRI detection

**Figure 3.**
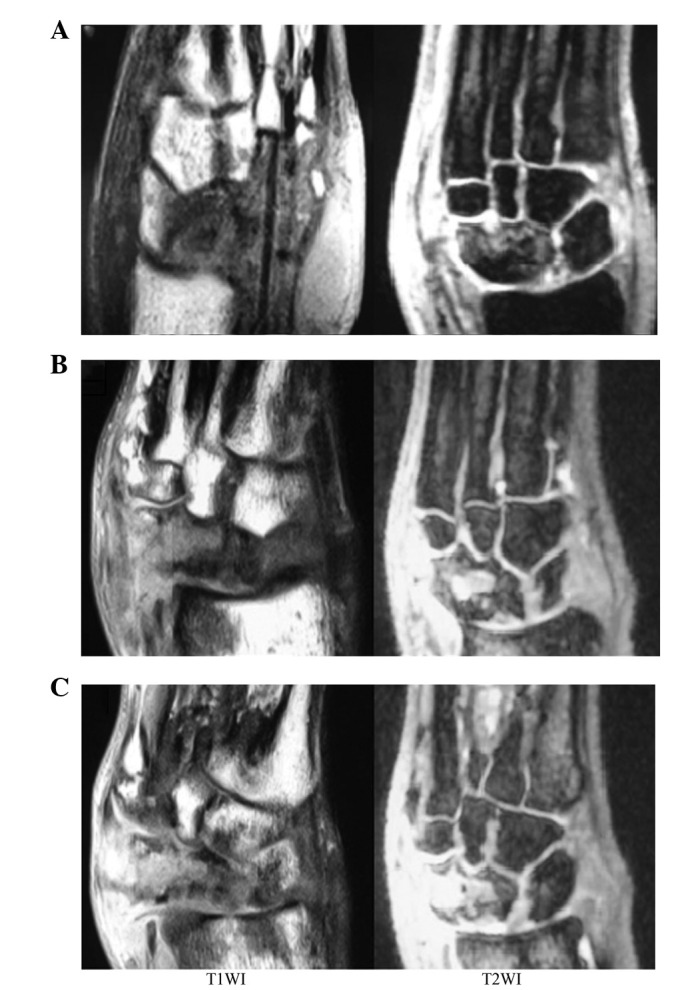
Images by magnetic resonance imaging (MRI) detection. (A) Images in week 3 after surgery. Hypointense foci were observed on T_1_-weighted images (T_1_WI) and disseminated punctate hyperintense foci were observed on T_2_WI. (B) Images in week 6 after surgery. Changes in the hypointense images on T_1_WI and hyperintense images on T_2_WI were observed. (C) Images in week 12 after surgery. A large area of hypointensity was detected on T_1_WI and a large area of hyperintensity was detected in the lunate on T_2_WI, within which disseminated areas of punctate hypointensity (necrotic bone chips) were embedded.

At 3 weeks after surgery, hypointense focus-like images on T_1_-weighted images (T_1_WI) and disseminated punctate hyperintense images on T_2_WI were observed. At 6 weeks after surgery, changes in the hypointense and hyperintense images were detected. At 12 weeks after surgery, large areas of hypointensity and hyperintensity were captured on T_1_WI and T_2_WI, respectively. Areas of diffuse punctate hypointensity were embedded in the large area of hyperintensity (Fig. 3).

### Histomorphological observation: gross sample observation

At 6 weeks after surgery, the lunate presented destroyed sclerotin on the surface, a smaller volume compared with the control, a dull articular surface without luster and a marked decrease in the cancellous bone density. At 12 weeks after surgery, a large area of dead bones with crisp texture was detected on the coronal section of the lunate. Furthermore, a number of cancellous bones were observed under the cartilage surface, as a result of osteosclerosis.

### Observation under a light microscope

**Figure 4.**
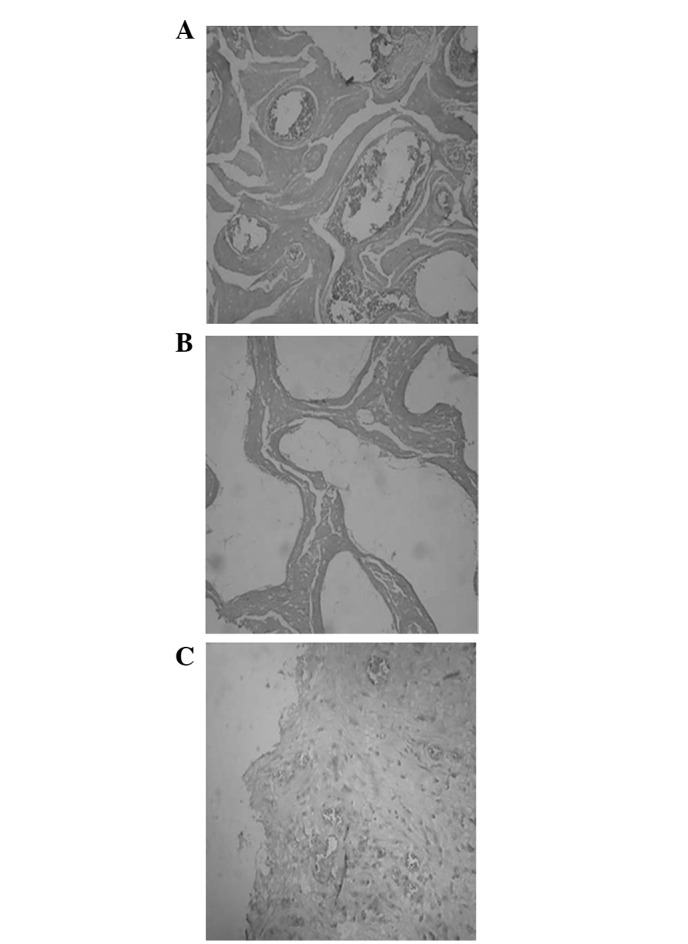
Results of histomorphological examination. (A) Image of the un-intervened lunate in week 3 after surgery. Abundant trabecular bones, full bone lacuna and plenty of capillary vessels were observed. (B) Image in week 6 after surgery. Sparseness of the trabecular bones, disappearance of the vessels, loss of calcium salt and disappearance of a large area of osteocytes were observed. (C) Image in week 12 after surgery. Fibroplasia was observed around the necrotic bones.

At 3 weeks after surgery, the trabecular bones appeared thinner, a number of bone lacunas became vacant and vessel density decreased. The marrow cavity bled and neither osteoblasts nor osteoclasts were detected. At 6 weeks after surgery, the trabecular bones became sparse and fractured, calcium salt was lost and a large area of osteocytes disappeared. More than 50% of the bone lacunas became vacant and inflammatory cell infiltration was observed. At 12 weeks after surgery, the trabecular bones fractured, all bone lacunas were empty and fibroplasia was identified around the trabecular bones. By contrast, the corresponding healthy lunate presented normal bone tissues, in which abundant trabecular bones, full lacunas and abundant capillary vessels were observed (Fig. 4).

## Discussion

The current methods used for the preparation of models of osteonecrosis include the following: i) hormone induction: Zhao *et al* used hormones and endotoxins to induce femur head necrosis in rabbits (13). They found that satisfactory necrosis was achieved within a short time (7). ii) Blood vessel ligation: Nishino *et al* prepared models with osteonecrosis through artificial dislocation of the hip joint and ligations of the medial and lateral femoral circumflex arteries and veins (14). These authors identified that dislocation of the joint or ligations of the blood vessels alone do not lead to osteonecrosis, and osteonecrosis only occurs when blood flow is at least 20% lower than that in the healthy control. iii) Osteotomy: Gong *et al* established models with femur head ischemic necrosis through osteotomy and apparent osteonecrosis was obtained two months after surgery (15). iv) Liquid nitrogen freezing: Yang *et al* established models with femur head necrosis via liquid nitrogen imbibition (16). These authors identified that the whole femur head presented complete necrosis with empty bone lacunas and necrotic tissue fragments filling the pulp chamber. Aside from the aforementioned techniques, methods used for the preparation of models of osteonecrosis also include local cell inactivation, the microwave method and acid base destruction.

Bone absorption and formation are closely associated with the roles of blood vessels in the bone (17). During bone growth and repair, bone formation is initiated and supported by blood vessels. Osteoblasts differentiate and proliferate around the vessel, arrange along the vascular endothelium and then excrete osteoids in a direction away from the vessel. When the osteoblasts mature and develop into osteocytes, the newly formed bones deposit around the vessel. In the current study, liquid nitrogen freezing, isolation of the lunate from its surrounding tissues and destruction of the blood supply for the lunate were adopted, to prepare models of lunatomalacia. Sparseness and disruption of the trabecular bones, loss of calcium salt, disappearance of a large area of osteocytes and infiltration of inflammatory cells were found at 6 weeks after surgery. Systemic or local intravascular coagulation is the last common pathway in the pathogenetic process of osteonecrosis caused by various factors (16). Liquid nitrogen freezing instantly leads to vasospasm and embolism in the lunate due to the small volume of the lunate and extenuation of the blood vessel supply running in it. As a result, vascular permeability is enhanced, vascular endothelial cells are damaged and intravascular coagulation is disseminated, thereby causing hemorrhage and ischemic reperfusion injury after rewarming (18,19). The vascular injury in the lunate combined with the isolation of the lunate from its surrounding soft tissues inhibited the spontaneous repair reactions of the lunate.

According to the improved Lichtman staging criteria formulated by Allan *et al*(8), Kienböck’s disease is divided into four stages. At stage I, pain, loss of strength and motion limitation at the wrist may occur. No abnormality is detected by X-ray in the majority of cases but a number of abnormal changes may be identified by MRI. At stage II, the bone density and fragments in the lunate increase; however, its volume, shape and anatomic associations with adjacent bones do not change. At stage III, the lunate begins to collapse. At stage IIIa, the location and correspondence of the wrist bones are normal. At stage IIIb, the joint space between the lunate and scaphoid widens. Palmar flexion of the scaphoid, an angle between the scaphoid and radius of >60° and ulnar deviation of the triangular bone are observed. Finally, at stage IV, the lunate collapses and fragments, the capitate bone shifts proximally, osteoarthritis occurs between the intercarpal joints, joint surfaces become coarse and uneven, joint spaces narrow, osteophytes are formed and the bones are hardened and degenerated cystically. In the present study, hypointense and disseminated hyperintense images were captured on T_1_WI and T_2_WI, respectively, at 3 weeks after surgery. However, such changes were not detected by X-ray or CT at the same time point. These results indicate that MRI possesses a hyper-sensitivity to early lunatomalacia. At 12 weeks after surgery, a hyperintense area in which areas of punctate hypointensity were embedded was observed on T_2_WI. The hyperintense area represented watery changes caused by inflammatory and repair reactions and the hypointense images in the area were necrotic bone chips.

Lunatomalacia is an independent clinical disease. Thus far, little is known about the cause of this disease. Factors including nutrient artery injury of the lunate, venous embolism, ulnar variation, morphologic abnormality of the lunate, rheumatoid arthritis, sicklemia, cerebral palsy, application of non-steroidal drugs and mechanical injuries are thought to be correlated with the incidence of lunatomalacia. Lunatomalacia is currently accepted as the consequence of the combined actions of various factors and its essence is bone avascular necrosis, in which anatomical variation may be the causative factor and acute and chronic injuries and inflammation may be its primary factor (20,21).

To date, studies on animal models with lunatomalacia have rarely been reported. Various methods have been used for model preparation. These methods have attempted to simulate the possible causes and pathological processes of osteonecrosis to provide an experimental basis for further studies on this disease. Sunagawa *et al* successfully prepared dog models with discontinuity between the scaphoid and lunate by resorting to artificial scapholunatum fractures and liquid nitrogen freezing (7). However, none of these methods reflect the pathological process of osteonecrosis completely. Therefore, the selection of proper experimental models for different purposes is of great practical significance. In this study, animal models of osteonecrosis were prepared by the destruction of blood supply to the lunate and liquid nitrogen freezing. Compared with other methods, this method has a number of advantages. First, it achieves a high success rate with reliable osteonecrosis and a short pathological process. Second, it is easy and convenient to perform. Third, it duplicates the primary pathological process of lunatomalacia, although not completely. Finally, no residue of drugs or chemical materials is left and the basic properties of the bone are maintained. Based on the advantages of this method in model preparation, the present study is expected to provide a good platform for further remedial studies on lunatomalacia.

## References

[1] Jones JP (1993). Fat embolism, intravascular coagulation and osteonecrosis. Clin Orthop Relat Res.

[2] Nakamura T, Matsumoto T, Nishino M, Tomita K, Kadoya M (1997). Early magnetic resonance imaging and histologic findings in a model of femoral head necrosis. Clin Orthop Relat Res.

[3] Ledoux P, Lamblin D, Wuilbaut A, Schuind F (2008). A finite-element analysis of Kienbock’s disease. J Hand Surg Eur Vol.

[4] Kienböck R (1910). Uber traumaische malazie des mondbeins und ihre folgezustande: Entartungs for men und kompressionsfrakturen. Fortschritte Rontgenstrahlen.

[5] Gelberman RH, Bauman TD, Menon J, Akeson WH (1980). The vascularity of the lunate bone and Kienböck’s disease. J Hand Surg Am.

[6] Kristensen SS, Søballe K (1987). Kienböck’s disease - the influence of arthrosis on ulnar variance measurements. J Hand Surg Br.

[7] Sunagawa T, Bishop AT, Muramatsu K (2000). Role of conventional and vascularized bone grafts in scaphoid nonunion with avascular necrosis: A canine experimental study. J Hand Surg Am.

[8] Allan CH, Joshi A, Lichtman DM (2001). Kienbock’s disease: diagnosis and treatment. J Am Acad Orthop Surg.

[9] Razemon JP (1982). Pathological study of Kienboeck’s disease. Ann Chir Main.

[10] Mirabello SC, Rosenthal DL, Smith RJ (1987). Correlation of clinical and radiographic findings in Kienböck’s disease. J Hand Surg Am.

[11] Kmano M, Koshimune M, Toyama M, Kazuki K (1991). Palmar plating system for Colles’ fractures - a preliminary report. J Hand Surg Am.

[12] Lichtman DM, Degnan GG (1993). Staging and its use in the determination of treatment modalities for Kienböck’s disease. Hand Clin.

[13] Zhao J, Lu S, Xue Z, Deng Q (2007). Establish and evaluate animal models of steriod-induced osteonecrosis of the femoral head. Chin J Bone Tumor & Bone Disease.

[14] Nishino M, Matsumoto T, Nakamura T, Tomita K (1997). Pathological and hemodynamic study in a new model of femoral head necrosis following traumatic dislocation. Arch Orthop Trauma Surg.

[15] Gong X, Lu L (2005). The development of Treating of Lunatomalacia. Foreign Medical Sciences (Section of Orthopaedics).

[16] Yang S, Yang C, Xu W, Li J, Zhang Y (2001). Avascular necrosisi of the Femoral Head Prouced in Rabbits by Freezing. Orthopedic J China (Chin).

[17] Beredjiklian PK (2009). Kienböck’s disease. J Hand Surg Am.

[18] Goldfarb CA, Hsu J, Gelberman RH, Boyer MI (2003). The Lichtman classification for Kienböck’s disease: an assessment of reliability. J Hand Surg Am.

[19] Bain GI, Begg M (2006). Arthroscopic assessment and classification of Kienbock’s disease. Tech Hand Up Extrem Surg.

[20] Keith PP, Nuttall D, Trail I (2004). Long-term outcome of nonsurgically managed Kienböck’s disease. J Hand Surg Am.

[21] Moran SL, Cooney WP, Berger RA, Bishop AT, Shin AY (2005). The use of the 4+5 extensor compartmental vascularized bone graft for the treatment of Kienböck’s disease. J Hand Surg Am.

